# Supplementary material for: Pyruvate Dehydrogenase Kinase 1 inhibition mediated oxidative phosphorylation enhancement in cartilage promotes osteoarthritis progression
Source: BMC Musculoskelet Disord. 2023 Jul 20;24:597. doi: 10.1186/s12891-023-06585-6 (PMC10357736; doi:10.1186/s12891-023-06585-6)
Supplement: Supplementary file 1 — Additional file 1. [file 12891_2023_6585_MOESM1_ESM.zip › BP.Gsea.1653623667859/GOBP_NUCLEOLUS_ORGANIZATION.html]

Details for gene set GOBP\_NUCLEOLUS\_ORGANIZATION[GSEA]

|  || Dataset | 55457\_collapsed\_to\_symbols.cls.cls #OA\_versus\_normal.cls.cls #OA\_versus\_normal\_repos |
| Phenotype | cls.cls#OA\_versus\_normal\_repos |
| Upregulated in class | normal |
| GeneSet | GOBP\_NUCLEOLUS\_ORGANIZATION |
| Enrichment Score (ES) | -0.666821 |
| Normalized Enrichment Score (NES) | -1.8950067 |
| Nominal p-value | 0.0 |
| FDR q-value | 0.055705044 |
| FWER p-Value | 0.735 |
Table: GSEA Results Summary

  

Fig 1: Enrichment plot: GOBP\_NUCLEOLUS\_ORGANIZATION      
 Profile of the Running ES Score & Positions of GeneSet Members on the Rank Ordered List

  

| SYMBOL | TITLE | RANK IN GENE LIST | RANK METRIC SCORE | RUNNING ES | CORE ENRICHMENT || 1 | RRP8 | ribosomal RNA processing 8 [Source:HGNC Symbol;Acc:HGNC:29030] | 1673 | 0.402 | -0.0640 | No |
| 2 | EMG1 | EMG1 N1-specific pseudouridine methyltransferase [Source:HGNC Symbol;Acc:HGNC:16912] | 1688 | 0.401 | 0.0004 | No |
| 3 | PHF2 | PHD finger protein 2 [Source:HGNC Symbol;Acc:HGNC:8920] | 5332 | 0.045 | -0.2749 | No |
| 4 | RPS19 | ribosomal protein S19 [Source:HGNC Symbol;Acc:HGNC:10402] | 5952 | -0.002 | -0.3227 | No |
| 5 | MACROH2A1 | macroH2A.1 histone [Source:HGNC Symbol;Acc:HGNC:4740] | 5953 | -0.002 | -0.3225 | No |
| 6 | SUV39H1 | suppressor of variegation 3-9 homolog 1 [Source:HGNC Symbol;Acc:HGNC:11479] | 6028 | -0.007 | -0.3271 | No |
| 7 | SIRT2 | sirtuin 2 [Source:HGNC Symbol;Acc:HGNC:10886] | 6279 | -0.026 | -0.3423 | No |
| 8 | SIRT1 | sirtuin 1 [Source:HGNC Symbol;Acc:HGNC:14929] | 8947 | -0.227 | -0.5121 | No |
| 9 | NOLC1 | nucleolar and coiled-body phosphoprotein 1 [Source:HGNC Symbol;Acc:HGNC:15608] | 10942 | -0.423 | -0.5975 | Yes |
| 10 | USP36 | ubiquitin specific peptidase 36 [Source:HGNC Symbol;Acc:HGNC:20062] | 11560 | -0.508 | -0.5623 | Yes |
| 11 | PHF8 | PHD finger protein 8 [Source:HGNC Symbol;Acc:HGNC:20672] | 11592 | -0.514 | -0.4806 | Yes |
| 12 | RRN3 | "RRN3 homolog, RNA polymerase I transcription factor [Source:HGNC Symbol;Acc:HGNC:30346]" | 11762 | -0.543 | -0.4049 | Yes |
| 13 | DDX11 | DEAD/H-box helicase 11 [Source:HGNC Symbol;Acc:HGNC:2736] | 12607 | -0.803 | -0.3391 | Yes |
| 14 | POLR1B | RNA polymerase I subunit B [Source:HGNC Symbol;Acc:HGNC:20454] | 12841 | -1.045 | -0.1863 | Yes |
| 15 | BAZ2A | bromodomain adjacent to zinc finger domain 2A [Source:HGNC Symbol;Acc:HGNC:962] | 12879 | -1.166 | 0.0016 | Yes |
Table: GSEA details [plain text format]

  

Fig 2: GOBP\_NUCLEOLUS\_ORGANIZATION      
 Blue-Pink O' Gram in the Space of the Analyzed GeneSet

  

Fig 3: GOBP\_NUCLEOLUS\_ORGANIZATION: Random ES distribution      
 Gene set null distribution of ES for **GOBP\_NUCLEOLUS\_ORGANIZATION**
